# Paeonol and Its Metabolites Alleviate LPS/D-GalN-Induced Acute Liver Injury in Mice: Potential Involvement of NDUFS7 and Macrophage Mitochondrial Function

**DOI:** 10.3390/antiox15091079

**Published:** 2026-08-28

**Authors:** Xin-Ru Lyu, Si-Tao Xu, Na Su, Min Lin, Zi-Ya Zhao, Zi-Han Xu, Xiang Li, Zhi-Hui Lu, Tong-Tong Wei, Shi-Yu Zhang, Qiang Fu, Guang-Ji Wang, Ying Peng, Jian-Guo Sun

**Affiliations:** 1 Jiangsu Provincial Key Laboratory of Drug Metabolism and Pharmacokinetics, China Pharmaceutical University, Nanjing 210009, China; 3123074303@stu.cpu.edu.cn (X.-R.L.); 3123074310@stu.cpu.edu.cn (S.-T.X.); na.su@lglab.ac.cn (N.S.); 3224071792@stu.cpu.edu.cn (M.L.); 3222071688@stu.cpu.edu.cn (Z.-Y.Z.); 3225071719@stu.cpu.edu.cn (Z.-H.X.); 3324071899@stu.cpu.edu.cn (X.L.); 3225071715@stu.cpu.edu.cn (Z.-H.L.); 3325071807@stu.cpu.edu.cn (T.-T.W.); 3325071721@stu.cpu.edu.cn (S.-Y.Z.); gjwang@cpu.edu.cn (G.-J.W.); 2 State Key Laboratory of Natural Medicines, China Pharmaceutical University, Nanjing 210009, China; 3 Department of Traditional Chinese Medicine and Pharmacology, School of Traditional Chinese Materia Medica, China Pharmaceutical University, Nanjing 211198, China; fuqiang@cpu.edu.cn; 4 School of Basic Medicine and Clinical Pharmacy, China Pharmaceutical University, Nanjing 211198, China

**Keywords:** acute liver injury, macrophages, mitochondria, mitochondrial complex I, NDUFS7, oxidative stress, paeonol, reactive oxygen species

## Abstract

Acute liver injury (ALI) is a severe clinical syndrome characterized by systemic inflammation and mitochondrial oxidative stress. This study investigated the hepatoprotective effects of paeonol (PA) in LPS/D-GalN-induced ALI and explored the potential involvement of macrophage mitochondrial function and NDUFS7. Proteomic analysis showed that the expression of mitochondrial respiratory chain-related proteins was suppressed in the liver of ALI mice, while PA treatment was associated with increased expression of several of these proteins. Integrated metabolomic analysis further indicated that PA modulated metabolites associated with energy metabolism and redox processes. In vitro experiments suggested that inflammatory RAW264.7 macrophages contributed to hepatocyte injury, whereas PA attenuated inflammatory responses and reduced macrophage-mediated injury to AML12 cells. In RAW264.7 cells, PA attenuated ROS accumulation and modulated multiple mitochondrial functional parameters, including mitochondrial membrane potential, ATP levels, the NAD^+^/NADH ratio, and complex I activity. Metabolic studies in human liver microsomes and primary human hepatocytes identified three major PA glucuronide metabolite products with five possible structural assignments. Molecular docking and microscale thermophoresis further indicated that several proposed PA metabolite structures exhibited more favorable predicted interactions and stronger binding to recombinant NDUFS7 than the parent PA. Collectively, these findings support the hepatoprotective effects of PA and its modulation of macrophage mitochondrial function, as well as the potential involvement of PA metabolites and NDUFS7, while further functional validation is required to establish their causal roles.

## 1. Introduction

Acute liver injury (ALI) is a severe clinical syndrome characterized by rapid progression and extensive hepatocyte death [1]. ALI is an important cause of acute liver failure and can progress to a life-threatening condition within days or weeks [2]. Current treatment options are largely supportive, with liver transplantation remaining the definitive treatment for patients with severe acute liver failure [3,4]. Therefore, therapeutic strategies that reduce hepatocyte injury and prevent disease progression are urgently needed.

Beyond inflammation, oxidative stress is increasingly recognized as an important driver of hepatocyte injury in ALI. Excessive generation of reactive oxygen species (ROS), particularly in association with mitochondrial electron transport chain dysfunction, can overwhelm endogenous antioxidant defenses such as the Nrf2 signaling pathway and further amplify inflammatory responses, thereby contributing to oxidative and inflammatory injury [5]. Mitochondrial complex I is an important component of the electron transport chain and is closely associated with cellular energy metabolism and ROS generation. Thus, mitochondrial dysfunction and redox alterations represent potentially important processes in the pathogenesis of ALI.

Clinical studies have shown that inflammatory markers are closely associated with the progression of liver injury [6]. The lipopolysaccharide (LPS)/D-galactosamine (D-GalN)-induced mouse model is widely used to investigate inflammatory mechanisms of ALI [7]. In this model, increased production of pro-inflammatory mediators, including tumor necrosis factor-α (TNF-α), interleukin-1β (IL-1β), interleukin-6 (IL-6), inducible nitric oxide synthase (iNOS) and cyclooxygenase-2 (COX-2), is associated with LPS/D-GalN-induced hepatic damage. For example, TNF-α can enhance the inflammatory response and contribute to hepatocyte apoptosis [8]. Macrophages play an important role in this process [9]. During the early stage of ALI, macrophages can acquire an inflammatory phenotype, thereby promoting the propagation and persistence of inflammation through the production of pro-inflammatory cytokines and mediators. Macrophages can also contribute to hepatocyte death through extracellular traps [10] and secretory vesicles [11]. Further, the hypoxic environment associated with severe liver injury can promote metabolic reprogramming of macrophages toward aerobic glycolysis [12]. Inflammatory stimulation also alters macrophage metabolism, and changes in the tricarboxylic acid (TCA) cycle and mitochondrial respiration are closely associated with inflammatory responses [13]. Targeting inflammatory macrophages has therefore emerged as a potential therapeutic strategy. For example, artificial apoptotic cells loaded with itaconic acid have shown beneficial effects against acetaminophen-induced acute liver failure and subsequent liver reinjury [14].

Paeonol (PA) is a phenolic compound mainly isolated from the root bark or cortex of *Paeonia* species and exhibits various pharmacological activities, including antipyretic, anti-inflammatory, antibacterial and analgesic effects. PA is available in several pharmaceutical formulations, including tablets, injections, and ointments. Oral and injectable preparations are mainly used to relieve pain and inflammation associated with dysmenorrhea and rheumatoid arthritis, whereas ointments are mainly used for the treatment of various skin diseases [15]. PA is also an important bioactive component of Guizhi Fuling Pills, a classic Chinese medicine prescription with anti-inflammatory activity and reported beneficial effects in hepatic fibrosis [16] and liver damage [17]. Previous studies suggest that PA may exert protective effects by attenuating oxidative stress and hepatic inflammation [18]. PA has also been reported to modulate mitochondrial function, including attenuation of glucose-induced mitochondrial damage, promotion of mitochondrial fusion and reduction in mitochondria-derived oxidative stress [19].

However, the mechanisms underlying the effects of PA in ALI remain incompletely understood. Previous studies have suggested that PA metabolites may contribute to its anti-inflammatory effects [20]. In addition, our previous human pharmacokinetic study of Guizhi Fuling Capsule identified multiple PA-related metabolites in human plasma and semi-quantitatively characterized their pharmacokinetic profiles using LC-MS/MS analysis [21]. Nevertheless, the potential involvement of PA metabolites in mitochondrial regulation and their molecular interactions remains unclear. Therefore, in the present study, we used an LPS/D-GalN-induced mouse model of ALI together with proteomic, metabolomic, cellular, and molecular interaction analyses to investigate the hepatoprotective effects of PA and to explore the potential involvement of macrophage mitochondrial function, PA metabolites, and NDUFS7.

## 2. Materials and Methods

### 2.1. Chemicals and Reagents

PA (Lot No. H811202) was purchased from Macklin (Shanghai, China); lipopolysaccharide (LPS, Lot No. L2880) and D-galactosamine hydrochloride (D-GalN, Lot No. G1639) were purchased from Sigma (St. Louis, MO, USA); acetonitrile and methanol were purchased from Merck (Darmstadt, Germany), and all other reagents were of analytical grade.

### 2.2. Instruments

High-performance liquid chromatography system (Shimadzu, Kyoto, Japan) coupled with a quadrupole tandem mass spectrometer (SCIEX, Framingham, MA, USA); Powerwave X-type microplate reader (BioTek Instruments, Winooski, VT, USA); IEC low temperature high-speed centrifuge (Thermo Fisher Scientific, Waltham, MA, USA); Sorvall Biofuge Stratos high-speed refrigerated centrifuge (Thermo Fisher Scientific, Waltham, MA, USA); Milli-Q Gradient A10 ultrapure water system (Millipore, Burlington, MA, USA); Minispin small high-speed centrifuge (Eppendorf, Hamburg, Germany); SynergyH1 full-function microplate detector (BioTek Instruments, Winooski, VT, USA); AUW 120D and AW120 electronic analytical balances (Shimadzu, Kyoto, Japan); vortex mixer (GENIE VORTEX-2, Scientific Industries, Bohemia, NY, USA); Mini-PROTEAN^®^ Tetra Cell (Bio-Rad Laboratories, Hercules, CA, USA); ChemiDoc XRS+ Gel Imaging System (Bio-Rad Laboratories, Hercules, CA, USA).

### 2.3. Cell Culture

RAW264.7 and AML12 cells were obtained from the cell collection of the Key Lab of Drug Metabolism and Pharmacokinetics, China Pharmaceutical University. The cell lines are registered in Cellosaurus with the accession numbers CVCL_0493 for RAW264.7 and CVCL_0140 for AML12, respectively. RAW264.7 cells were cultured in high-glucose DMEM (C11995500BT, Gibco, Grand Island, NY, USA) supplemented with 10% FBS (Gibco, Grand Island, NY, USA) and 1% penicillin-streptomycin (15140122, Gibco, Grand Island, NY, USA), while AML12 cells were cultured in AML12-specific medium (CM-0602, Procell, Wuhan, China). The cells were maintained in a conventional incubator at 37 °C, 5% CO_2_, and 90% relative humidity. Cell culture consumables were purchased from Corning Costar (Corning, NY, USA). To evaluate the potential cytotoxicity of PA at the concentrations used in RAW264.7 cell experiments, cell viability was assessed using a Cell Counting Kit-8 (CCK-8; C0039, Beyotime, Shanghai, China). RAW264.7 cells were treated with PA at concentrations of 0, 0.05, 0.1, 0.2, 0.5, and 1 mmol/L for 24 h, and cell viability was subsequently determined according to the manufacturer’s instructions by measuring the absorbance at 450 nm using a microplate reader.

### 2.4. Animals and Treatments

Thirty healthy male C57BL/6 mice (18–22 g) were provided by Shanghai Xipuer-bikai Experimental Animal Co., Ltd. (Shanghai, China; Experimental Animal Production License No. 202107477). The mice were housed under controlled conditions at a temperature of 22 ± 2 °C and relative humidity of 50 ± 10%. The animals were acclimatized for one week before the experiment. All animal experimental procedures were approved by the Animal Ethics Committee of China Pharmaceutical University (Ethical Review Acceptance No. 2021-04-010).

Mice were randomly divided into five groups (*n* = 6 per group): control, model, PA low-dose (PA-L), PA medium-dose (PA-M), and PA high-dose (PA-H) groups. Mice in the PA-L, PA-M and PA-H groups were administered PA suspended in CMC-Na at doses of 50, 100, and 200 mg/kg, respectively, by intragastric administration for 10 consecutive days, while mice in the control and model groups received CMC-Na vehicle. On day 10, 30 min after administration, mice in the control group received an intraperitoneal injection of normal saline (0.1 mL/10 g), whereas mice in the other groups received LPS (10 μg/kg) as well as D-GalN (200 mg/kg) by intraperitoneal injection. Body weight was recorded before and after model induction. At 6 h after LPS/D-GalN administration, blood samples were collected, followed by euthanasia. Serum and liver tissues were subsequently collected for further analyses.

### 2.5. Determination of Liver Damage Index and Inflammatory Markers

The levels of alanine aminotransferase (ALT, C009-2-1, Nanjing Jiancheng Bioengineering Institute, Nanjing, China), aspartate aminotransferase (AST, C010-2-1, Nanjing Jiancheng Bioengineering Institute, Nanjing, China), IL-6 (E-EL-M0044c, Elabscience Biotechnology Co., Ltd., Wuhan, China), IL-1β (E-EL-M0037c, Elabscience Biotechnology Co., Ltd., Wuhan, China), and TNF-α (E-EL-M3063, Elabscience Biotechnology Co., Ltd., Wuhan, China) in serum were measured according to the manufacturers’ instructions. After euthanasia, the livers were collected and weighed. A tissue section was collected from the same region of each liver and subjected to hematoxylin and eosin (HE) staining for histopathological examination using a DMI3000 B microscope (Leica, Wetzlar, Germany). Another portion of liver tissue was weighed, cut into pieces, and homogenized for determination of malondialdehyde (MDA) levels using an MDA assay kit (S0131M, Beyotime, Shanghai, China).

### 2.6. RNA Extraction and Real-Time PCR

RNA was extracted from liver tissues, RAW264.7 cells and AML12 cells using TRIzol (R401-01, Vazyme, Nanjing, China). After mixing with HiScript III Reverse Transcriptase SuperMix for qPCR (R323-01, Vazyme, Nanjing, China), samples were transferred to the T100 Thermal Cycler (Bio-Rad, Hercules, CA, USA), and reverse transcription was performed at 37 °C for 15 min and 85 °C for 5 s to obtain cDNA. The qPCR reaction mixture contained 1 μL of cDNA, 1 μL of forward primer, 1 μL of reverse primer, 7.5 μL of ChamQ SYBR qPCR Master Mix (Q311-02, Vazyme, Nanjing, China) and 4.5 μL of DEPC-treated water (R0022, Beyotime, Shanghai, China). Amplification was performed using CFX96^TM^ Real-Time System (Bio-Rad, Hercules, CA, USA) under the following cycling conditions: 95 °C for 10 s, 60 °C for 30 s, and then 72 °C for 30 s for 40 cycles. The primer sequences (Invitrogen, Waltham, MA, USA) are shown in Table 1.

### 2.7. Western Blot Analysis

Liver tissue samples (20 mg) were weighed from each group and lysed using radioimmunoprecipitation assay lysis buffer (RIPA Lysis Buffer, Beyotime, Shanghai, China) supplemented with phenylmethylsulfonyl fluoride (PMSF, Beyotime, Shanghai, China). Protein concentrations were determined using a BCA assay kit (P0009, Beyotime, Shanghai, China). Equal amounts of protein were separated by 10% SDS-PAGE using a Color PAGE Gel Rapid Preparation Kit (Epizyme Biotech, Shanghai, China) and transferred onto Immun-Blot^®^ PVDF membranes (Bio-Rad, Hercules, CA, USA). After blocking, the membranes were incubated with the respective primary antibodies overnight at 4 °C. The primary antibody against COX-2 (#12282) was purchased from Cell Signaling Technology (Danvers, MA, USA), while antibodies against iNOS (18985-1-AP), GAPDH (60004-1-Ig), and NDUFS7 (15728-1-AP) were purchased from Proteintech (Wuhan, China). The membranes were subsequently incubated with the appropriate secondary antibodies (BS12478 and BS13278, Bioworld Technology Co., Ltd., Bloomington, MN, USA) for 2 h. Immunoreactive bands were visualized using Clarity™ Western ECL Substrate (Bio-Rad, Hercules, CA, USA).

### 2.8. Proteomic Analysis

#### 2.8.1. Processing of Samples for Proteomics

Liver tissue samples (50 mg) were collected from control, model, and PA-H groups. Proteins were extracted using RIPA lysis buffer supplemented with PMSF, and the protein concentration was determined using a BCA assay kit. For each sample, 200 μg of protein was added to an ultrafiltration tube (VN01H02, Sartorius, Gottingen, Germany), and 8 mol/L urea solution was added until the total volume reached 200 μL. Samples were centrifuged at 14,000× *g* for 20 min. Subsequently, 200 μL urea solution was added to each tube, followed by centrifugation at 14,000× *g* for 20 min. Then, 200 μL UA buffer (containing 0.1 mol/L Tris and 8 mol/L urea) and 2 μL of 10 mmol/L dithiothreitol (DTT, Sigma-Aldrich, Hamburg, Germany) were added to the upper layer.

Samples were incubated in a water bath at 60 °C for 1 h and centrifuged at 14,000× *g* for 10 min. Subsequently, 200 μL UA buffer solution and 8 μL of 20 mmol/L iodoacetamide (IAM, Sigma-Aldrich, Hamburg, Germany) solution were added to the upper layer. Samples were incubated at room temperature for 45 min in the dark and centrifuged at 14,000× *g* for 20 min. 200 μL of 50 mmol/L ammonium bicarbonate solution was added to the upper layer and centrifuged at 14,000× *g* for 10 min. For each sample, 200 μL of 20 μg/mL trypsin (Sigma-Aldrich, Hamburg, Germany) solution was added to the upper ultrafiltration tube. The filtrate was collected and evaporated to dryness in a vacuum spin dryer (Speed-vac, Thermo Savant, Waltham, MA, USA), with the vacuum level set to 10 in manual-run mode. Each sample was then redissolved in 100 μL of 0.1% formic acid-water and centrifuged at 29,850× *g* for 5 min. 80 μL of supernatant was transferred into the glass inserts (5181-1270, Agilent, Santa Clara, CA, USA) for proteomic analysis. Proteomic detection methods were listed in Supporting Information.

#### 2.8.2. Proteomic Data Processing

After pre-injection of the mixed sample, the ion distribution was imported into SWATH Variable Window Calculator v1.1 (SCIEX, Framingham, MA, USA) for calculation. The results were imported into Analyst software (version 1.7.1, AB SCIEX, Framingham, MA, USA) to construct the SWATH method. SWATH data were processed using ProteinPilot software (version 5.0, AB SCIEX, Framingham, MA, USA) for identification against the NCBI mouse protein database. The results were imported into the SWATH^TM^ processing plug-in in PeakView software (version 1.2, AB SCIEX, Framingham, MA, USA) for further processing. Differential proteins were screened using a fold-change threshold greater than 1.5 and a *p* value less than 0.05. Data visualization and enrichment analyses were performed using the Bioinformatics platform (Shanghai NewCore Biotechnology Co., Ltd., Shanghai, China). The STRING database, together with Cytoscape software (version 3.10.3, Cytoscape Consortium, San Diego, CA, USA) [22] was used for statistical analysis and visualization of protein–protein interactions.

### 2.9. Molecular Docking and Microscale Thermophoresis (MST) Assay

The chemical structures of PA and its metabolites were prepared using Discovery Studio 2016 Client (BIOVIA, San Diego, CA, USA). The structure of mitochondrial complex I (PDB ID: 6ZTQ) was obtained from the Research Collaboratory for Structural Bioinformatics Protein Data Bank (RCSB PDB; https://doi.org/10.2210/pdb6ZTQ/pdb; accessed on 10 August 2026). Molecular docking was performed using iGEMDOCK v2.1 (National Chiao Tung University, Hsinchu, Taiwan, China). The binding site was defined based on HQH, the native ligand in the 6ZTQ structure, using the “By bounded ligand” option with a binding-site radius of 8.0 Å. For each ligand, two independent docking runs were performed with a population size of 200 and 70 generations, and two docking solutions (predicted binding poses) were generated in each run. Docking poses were evaluated using the GEMDOCK scoring function (Fitness = vdW + Hbond + Elec), where vdW, Hbond, and Elec represent van der Waals, hydrogen-bonding, and electrostatic energies, respectively. Lower (more negative) fitness values indicate more favorable predicted protein–ligand interactions. The predicted binding poses and interacting residues of PA and its metabolites were further compared with the HQH-binding region in the 6ZTQ structure. As an additional qualitative assessment of the docking procedure, HQH was re-docked into the defined binding site using the same docking parameters, and the resulting binding poses were compared with the native HQH-binding region in the original 6ZTQ structure (Figure S10D).

Microscale thermophoresis (MST) was used to measure the equilibrium dissociation constant (Kd) between recombinant mouse NADH dehydrogenase [ubiquinone] iron-sulfur protein 7 (NDUFS7; CSB-YP884070MO, Cusabio Technology LLC, Wuhan, China) and PA as well as its metabolites. The recombinant NDUFS7 corresponded to residues 36–224 of the mature protein, was expressed in yeast, and carried a His-tag. The assay was performed according to the instructions of the Monolith His-Tag Labeling Kit RED-tris-NTA 2nd Generation (Cat. No. MO-L018, NanoTemper Technologies, Munich, Germany). Samples containing recombinant NDUFS7 and different compounds were loaded into Monolith NT.115 series capillaries (MO-K022, NanoTemper Technologies, Munich, Germany). MST measurements were performed using a Monolith NT.115 Pico instrument (NanoTemper Technologies, Munich, Germany), and the Kd values were analyzed using MO Analysis software (version 2.2.4, NanoTemper Technologies, Munich, Germany).

### 2.10. Detection of Cytokines, Reactive Oxygen Species (ROS) and Adenosine Triphosphate (ATP) and Mitochondrial Membrane Potential in RAW264.7 Cells

RAW264.7 cells were cultured in 96-, 24- and 6-well plates and divided into five groups: control, model, PA low-dose (PA-L), PA medium-dose (PA-M), and PA high-dose (PA-H) groups. After incubation for 24 h, cells in the control and model groups were treated with culture medium containing 0.1% DMSO, while cells in the PA-L, PA-M, and PA-H groups were treated with PA at concentrations of 0.2, 0.5, and 1 mmol/L, respectively. After 2 h, LPS was added to the culture medium of the model and PA treatment groups at a final concentration of 100 ng/mL, while an equal volume of blank medium was added to the control group. The cells were then incubated for an additional 24 h. Subsequently, the culture medium was collected, and NO (S0021M, Beyotime, Shanghai, China), IL-6 (E-EL-M0044c, Elabscience Biotechnology Co., Ltd., Wuhan, China), and TNF-α (E-EL-M3063, Elabscience Biotechnology Co., Ltd., Wuhan, China) levels were measured using the respective assay kits. Cellular ROS (S0033M, Beyotime, Shanghai, China) and ATP (S0027, Beyotime, Shanghai, China) levels were determined according to the respective instructions. Mitochondria were isolated from RAW264.7 cells using a Mitochondria/Nuclei Isolation Kit (KGP850, KeyGEN BioTECH, Nanjing, China). Mitochondrial membrane potential was assessed using an Enhanced Mitochondrial Membrane Potential Assay Kit with JC-1 (C2003, Beyotime, Shanghai, China) according to the manufacturer’s instructions and expressed as the ratio of red to green fluorescence.

### 2.11. Detection of Cytokine Levels in AML12 Cells

AML12 cells were cultured in 6-well plates and divided into five groups: control, LPS, LPS-treated RAW264.7 (LR), LPS-treated RAW264.7 + PA (LR + PA) and PA + LPS-treated RAW264.7 (PAR) groups. After RAW264.7 cells in the model and PA-M groups were incubated for 24 h, the culture medium was collected and mixed with an equal volume of AML12 medium. The resulting media were added to AML12 cells in the LR and PAR groups, respectively. AML12 cells in the control group were cultured in mixed medium containing equal volumes of blank DMEM and AML12 medium, while cells in the LPS group were cultured in the same mixed medium containing 1 μg/mL LPS. For the LR + PA group, AML12 cells were directly treated with 0.5 mmol/L PA and cultured with an equal volume of supernatant from RAW264.7 cells in the model group mixed with AML12 medium. Subsequently, the culture medium was collected, and IL-6 (E-EL-M0044c, Elabscience Biotechnology Co., Ltd., Wuhan, China) and TNF-α (E-EL-M3063, Elabscience Biotechnology Co., Ltd., Wuhan, China) levels were measured using the respective assay kits.

### 2.12. Method for Determination of PA

#### 2.12.1. Liquid Chromatography Conditions

Chromatographic separation was performed using a Kromasil 100-5C18 column (150 × 2.1 mm, Nouryon, Bohus, Sweden) at 40 °C. The mobile phases consisted of 0.1% formic acid in water (A) and acetonitrile (B) with a flow rate of 0.3 mL/min. The total analysis time was 7.5 min. The gradient elution program was as follows: 0–0.5 min, 10% B, 0.5–2.0 min, 10% to 90% B, 2.0–5.5 min, 90% B, 5.5–6 min, 90% to 10% B, and 6–7.5 min, 10% B.

#### 2.12.2. Mass Spectrometry Conditions

An AB SCIEX API 4000 mass spectrometer (AB SCIEX, Framingham, MA, USA) equipped with electrospray ionization (ESI) was operated in positive ion multiple reaction monitoring (MRM) mode. The MRM transitions for PA and the internal standard were *m*/*z* 167.1 → 121.1 and *m*/*z* 409.2 → 228.2, respectively. The injection volume was 10 μL.

#### 2.12.3. PA Uptake by Cells

RAW264.7 cells were cultured in 6-well plates. After incubation for 24 h, cells were treated with PA at a concentration of 1 mmol/L. PA-containing medium was discarded at 1, 2, 4, 8, 12, and 24 h after administration. After washing the cells three times with PBS, 1 mL of ultrapure water was added, and the cells were subjected to three freeze–thaw cycles by freezing at −80 °C and thawing at room temperature for cell disruption. The protein content of each sample was determined using a BCA protein assay kit. A 50 μL aliquot of each lysed cell suspension was transferred to a new tube, and 200 μL of protein precipitant (methanol containing 0.5 ng/mL tamsulosin) was added. Each sample was centrifuged twice at 29,850× *g* for 5 min, and 100 μL of the supernatant was transferred into tubes for PA analysis by LC-MS.

#### 2.12.4. Subcellular Distribution of PA

RAW264.7 cells were cultured in cell culture dishes. After incubation for 24 h, cells were treated with PA at a concentration of 1 mmol/L. Cells were collected at 6, 12 and 24 h after administration, respectively. Mitochondrial, nuclear and cytoplasmic fractions were isolated according to the instructions of a Mitochondria/Nuclei Isolation Kit (KGP850, KeyGEN BioTECH, Nanjing, China). The protein content of each sample was measured using a BCA protein assay kit (P0009, Beyotime, Shanghai, China). To 50 μL of each sample, 200 μL of protein precipitant (methanol containing 0.5 ng/mL tamsulosin) was added, followed by centrifugation twice at 29,850× *g* for 5 min. Then, 100 μL of the supernatant was transferred into tubes for PA analysis by LC-MS.

### 2.13. Metabolomic Analysis

#### 2.13.1. Processing of Serum Samples for Metabolomics

Serum samples (50 μL) from mice in the control, model and PA-H groups were transferred into clean tubes. For each sample, 200 μL of precipitant solution (methanol containing 15 μg/mL [^13^C]-glutamine, Cambridge Isotope Laboratories, Inc., Tewksbury, MA, USA) was added, followed by vortexing for 5 min and centrifugation at 20,000× *g* for 10 min. Then, 200 μL of the supernatant was transferred to a new tube and evaporated to dryness using a vacuum spin dryer (Speed-vac, Thermo Savant, Waltham, MA, USA). Quality control (QC) samples were prepared by combining equal volumes of supernatant from all samples to monitor analytical stability. The dried samples were reconstituted in 100 μL of ultrapure water and centrifuged at 29,850× *g* for 10 min. Finally, 80 μL of the supernatant was transferred to the inner tube for injection analysis. Detailed metabolomics detection methods are provided in the Supporting Information.

#### 2.13.2. Processing of Cell Samples for Metabolomics

After washing the cells three times with PBS, 300 μL of ultrapure water was added, and the cells were subjected to three freeze–thaw cycles by freezing at −80 °C and thawing at room temperature for cell disruption. The protein concentration of each sample was determined using a BCA assay kit (P0009, Beyotime, Shanghai, China). The remaining samples were subjected to two sequential extractions with precipitant solution (methanol containing 15 μg/mL [^13^C]-glutamine), and the resulting supernatants were combined for subsequent analysis. A total of 700 μL of the combined supernatant was transferred to a new tube and evaporated to dryness using a vacuum spin dryer (Speed-vac, Thermo Savant, Waltham, MA, USA). Quality control samples were prepared by combining equal volumes of supernatant from all samples to monitor analytical stability. The dried samples were reconstituted in 100 μL of ultrapure water and centrifuged at 29,850× *g* for 10 min. Finally, 80 μL of the supernatant was transferred for injection analysis. Detailed metabolomics detection methods are provided in the Supporting Information.

#### 2.13.3. Metabolomics Data Analysis

Metabolomics data were processed using MultiQuant software (version 2.0, AB SCIEX, Framingham, MA, USA). Peak areas were normalized to the internal standard prior to statistical analysis. Multivariate statistical analyses, including principal component analysis (PCA), partial least squares-discriminant analysis (PLS-DA), and orthogonal partial least squares-discriminant analysis (OPLS-DA), were performed using SIMCA-P software (version 13.0, Umetrics AB, Umeå, Sweden). Differential metabolites were screened using a variable importance in projection (VIP) value > 1 and a *p* value < 0.05. Data visualization was performed using the Bioinformatics platform. Pathway and enrichment analyses of differential metabolites were performed using MetaboAnalyst (version 5.0, Xia Lab, McGill University, Montreal, QC, Canada).

### 2.14. Statistical Analysis

Data are presented as the mean ± SEM. The sample size (*n*) for each group is indicated in the figure legends. Differences between two groups were analyzed using an unpaired Student’s *t*-test, while comparisons among multiple groups were performed using one-way analysis of variance (ANOVA) followed by Bonferroni’s multiple-comparison test. A *p* value < 0.05 was considered statistically significant.

## 3. Results

### 3.1. PA Had a Hepatoprotective Effect on ALI Mice

**Figure 1:**
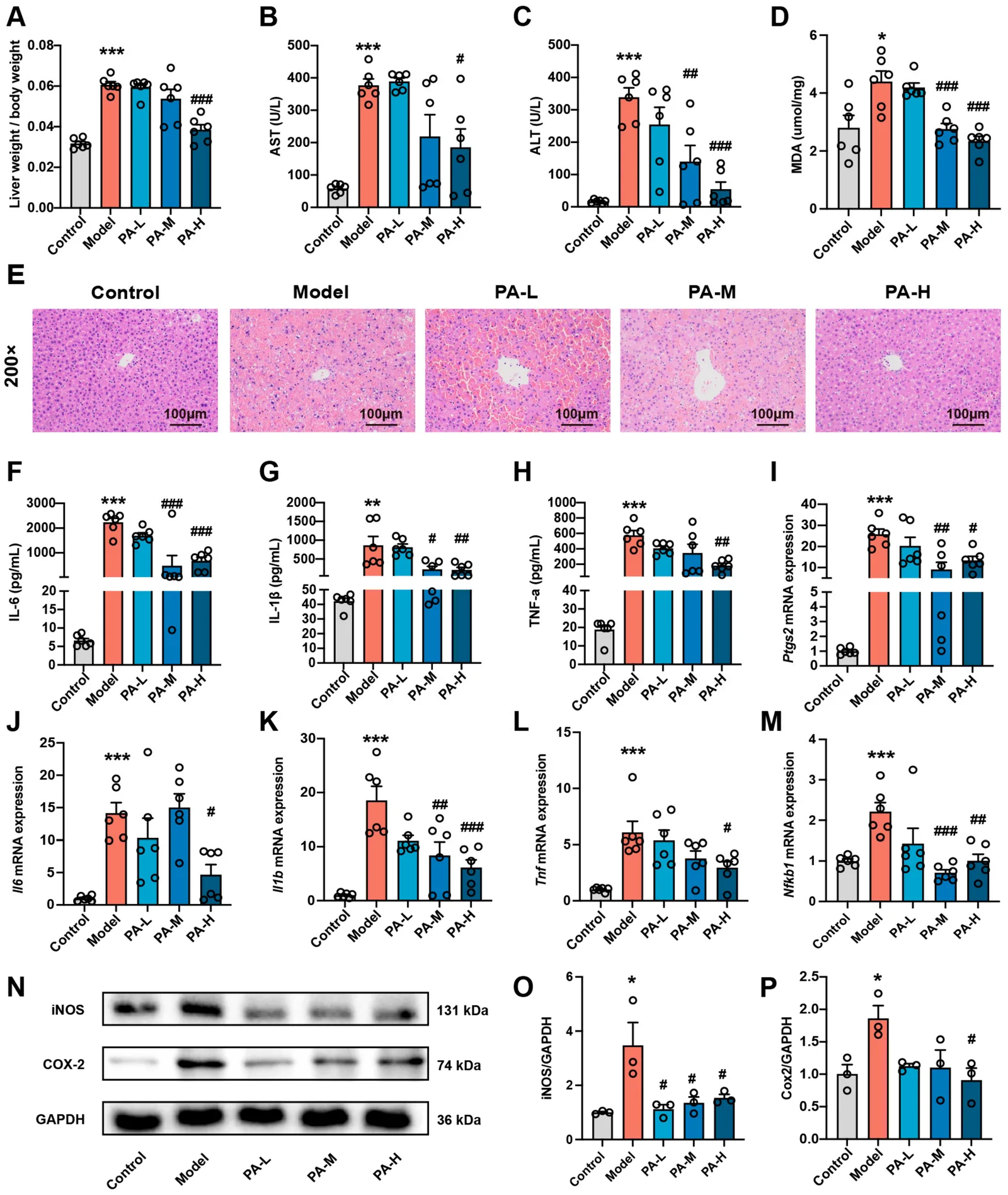
PA alleviated LPS/D-GalN-induced acute liver injury and inflammatory responses in mice. (**A**) The liver coefficient (liver weight/body weight) was increased following LPS/D-GalN challenge and reduced by PA treatment. (**B**,**C**) Serum AST and ALT levels and (**D**) hepatic MDA levels were increased in ALI mice and reduced following PA treatment. (**E**) Representative HE-stained liver sections showing the pathological changes in each group (scale bar = 100 μm; ×200). (**F**–**H**) Serum levels of IL-6, IL-1β, and TNF-α. (**I**–**M**) Hepatic mRNA expression levels of *Ptgs2*, *Il6*, *Il1b*, *Tnf*, and *Nfkb1*. PA treatment attenuated the increases in inflammatory cytokine levels and inflammatory gene expression induced by LPS/D-GalN. (**N**) Representative Western blot images of iNOS, COX-2, and GAPDH. (**O**,**P**) Quantitative analysis of iNOS and COX-2 protein levels. Data are presented as the mean ± SEM (**A**–**M**, *n* = 6; **O**,**P**, *n* = 3). Circles represent individual data points. * *p* < 0.05, ** *p* < 0.01, *** *p* < 0.001 vs. control group; # *p* < 0.05, ## *p* < 0.01, ### *p* < 0.001 vs. model group.

The liver coefficient, calculated based on the body and liver weights of mice in each group, was markedly increased in the model group compared with the control group and was reduced following PA treatment (Figure 1A). Serum ALT and AST levels and hepatic MDA levels in the model group were also markedly higher than those in the control group, whereas PA treatment significantly reduced these elevations (Figure 1B–D).

HE staining showed that hepatocytes in the control group were regularly arranged with normal morphology and clear structures, with no obvious inflammatory cell infiltration. In contrast, hepatocytes in the model group were disorganized, accompanied by extensive cell damage, hemorrhage, vacuolization and inflammatory cell infiltration. PA treatment alleviated these pathological changes in ALI mice (Figure 1E).

The levels of the inflammatory cytokines, IL-6, IL-1β and TNF-α, were markedly elevated in the model group and reduced following PA treatment (Figure 1F–H). Consistently, the mRNA levels of *Il6*, *Il1b* and *Tnf* were increased in the model group and attenuated by PA treatment (Figure 1J–L). The expression levels of *Ptgs2* and *Nfkb1* showed similar changes (Figure 1I–M). Western blot analysis further showed that iNOS and COX-2 protein levels were increased in ALI mice and reduced following PA treatment (Figure 1N–P).

### 3.2. High-Dose PA Regulated the Expression of Mitochondria-Related Proteins in ALI Mice

**Figure 2:**
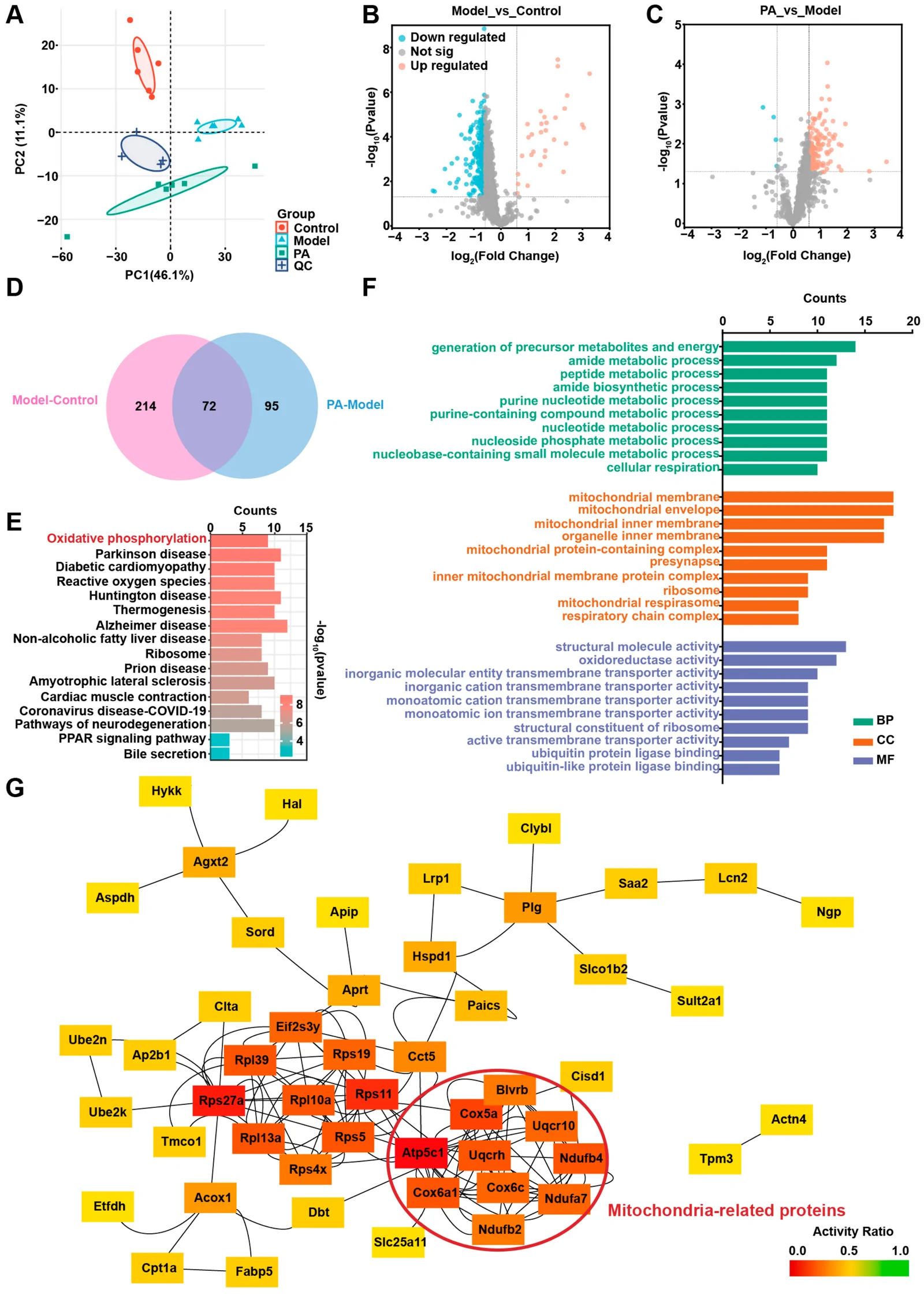
PA regulated the expression of mitochondria-related proteins in the livers of ALI mice. (**A**) PCA score plot of liver proteomic profiles from the control, model, and PA groups (confidence interval = 0.95). (**B**,**C**) Volcano plots of differential proteins in the model vs. control and PA vs. model comparisons. (**D**) Venn diagram showing 72 shared differential proteins between the two comparisons. (**E**) KEGG pathway enrichment analysis of the 72 shared differential proteins, with oxidative phosphorylation among the significantly enriched pathways. (**F**) GO enrichment analysis of the differential proteins, including biological process (BP), cellular component (CC), and molecular function (MF). (**G**) Protein–protein interaction network of the differential proteins, in which lines represent protein–protein interactions and the red circle highlights mitochondria-related proteins.

PCA of the proteomic data showed clear separation between the control and model groups, indicating substantial differences in protein expression. The PA group shifted toward the control group, indicating a partial shift in the liver proteomic profile following PA treatment. Quality control (QC) samples clustered tightly in the PCA score plot, indicating good analytical stability (Figure 2A). A total of 286 differential proteins were screened between the control and model groups, including 256 downregulated and 30 upregulated proteins in the model group (Figure 2B). Between the PA and model groups, 167 differential proteins were found, including 163 upregulated and 4 downregulated (Figure 2C). In addition, 72 proteins were shared between these comparisons (Figure 2D).

Kyoto Encyclopedia of Genes and Genomes (KEGG) pathway and Gene Ontology (GO) term enrichment analyses of the 72 differential proteins showed that the major changes were associated with oxidative phosphorylation, energy metabolism, cellular respiration, mitochondrial components, and oxidoreductase and transporter activities (Figure 2E,F). Metascape analysis yielded similar enrichment results, with metabolic processes being the most influenced parent pathways (Figure S1). In the protein–protein interaction network, the degree of each differential protein was calculated and ranked. The top 20 proteins were mainly associated with ribosomal and mitochondrial functions, with Cox5a, Ndufb4, Cox6a1, and Cox6c among the highly ranked proteins (Figure 2G). The abundance of mitochondria-related proteins was markedly reduced in the livers of ALI mice and increased following PA treatment (Figure S2).

### 3.3. PA Administration Regulated the Levels of Endogenous Metabolites in Serum of ALI Mice

**Figure 3:**
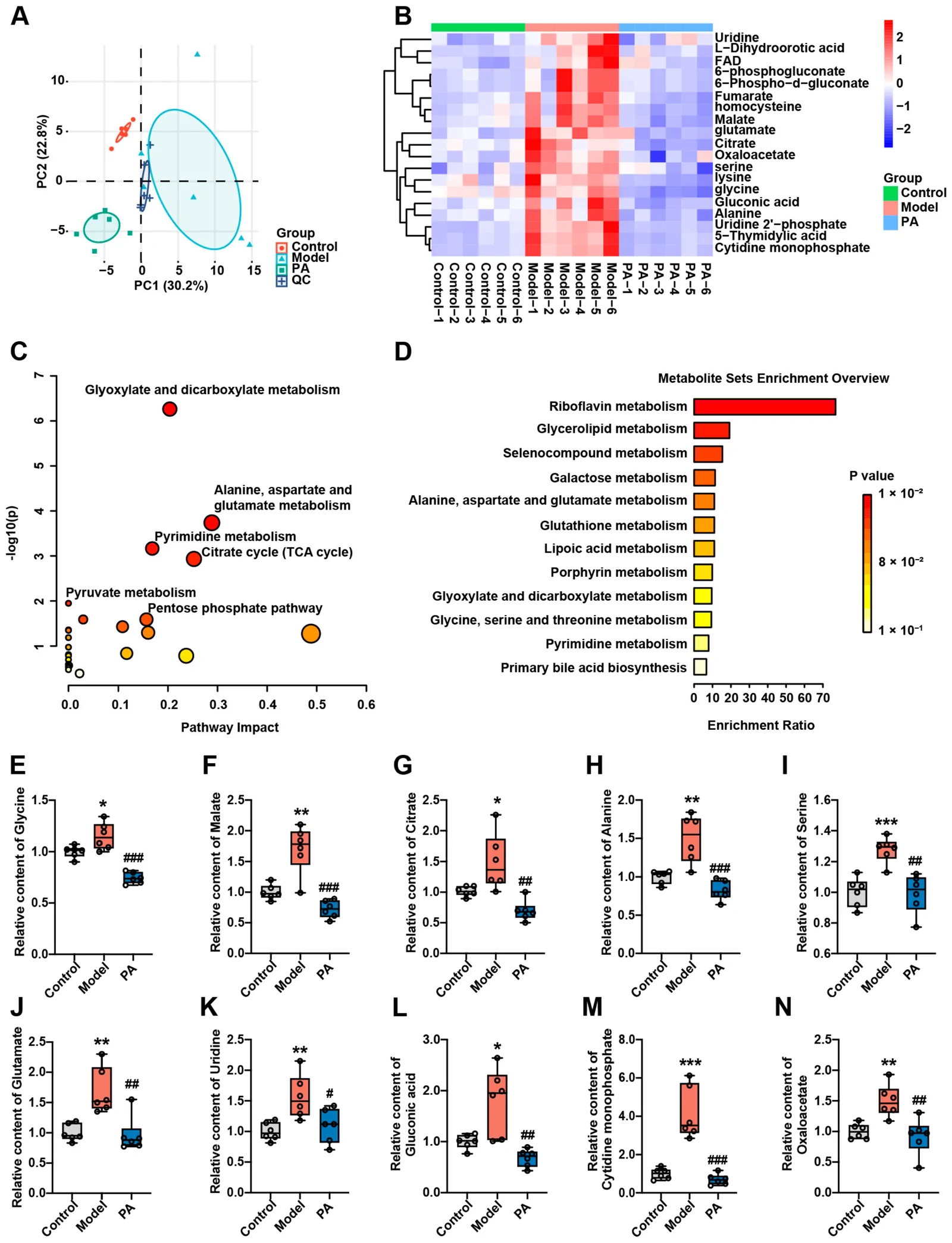
PA regulated serum metabolite levels in ALI mice. (**A**) PCA score plot of serum metabolomic profiles from the control, model, and PA groups (confidence interval = 0.95). (**B**) Heatmap of shared differential metabolites between the model vs. control and PA vs. model comparisons, with colors indicating relative metabolite levels. (**C**) Pathway analysis of the differential metabolites, in which circle size represents pathway impact and circle color indicates pathway significance. (**D**) Enrichment analysis of the differential metabolites, with colors indicating *p*-values. (**E**–**N**) Relative levels of representative differential metabolites; circles represent individual data points. Data are presented as mean ± SEM, *n* = 6, * *p* < 0.05, ** *p* < 0.01, *** *p* < 0.001 vs. control group; # *p* < 0.05, ## *p* < 0.01, ### *p* < 0.001 vs. model group.

PCA of the serum metabolomic data showed clear separation among the control, model, and PA groups. The model group was clearly separated from the control group, whereas the PA group shifted toward the control group. The QC samples clustered closely in the PCA plot, indicating good analytical stability (Figure 3A). Differential endogenous metabolites shared between the model vs. control and PA vs. model comparisons were selected for further analysis. Heatmap analysis showed distinct changes in the levels of differential endogenous metabolites among the control, model, and PA groups (Figure 3B). Pathway analysis showed that alanine, aspartate and glutamate metabolism, pyrimidine metabolism and citrate cycle (tricarboxylic acid cycle, TCA cycle) were notably affected (Figure 3C), while enrichment analysis highlighted riboflavin metabolism (Figure 3D). The levels of related differential endogenous metabolites were increased in the model group and reduced following PA treatment (Figure 3E–N).

### 3.4. PA Indirectly Alleviated Inflammatory Responses in Hepatocytes by Regulating RAW264.7 Cells

**Figure 4:**
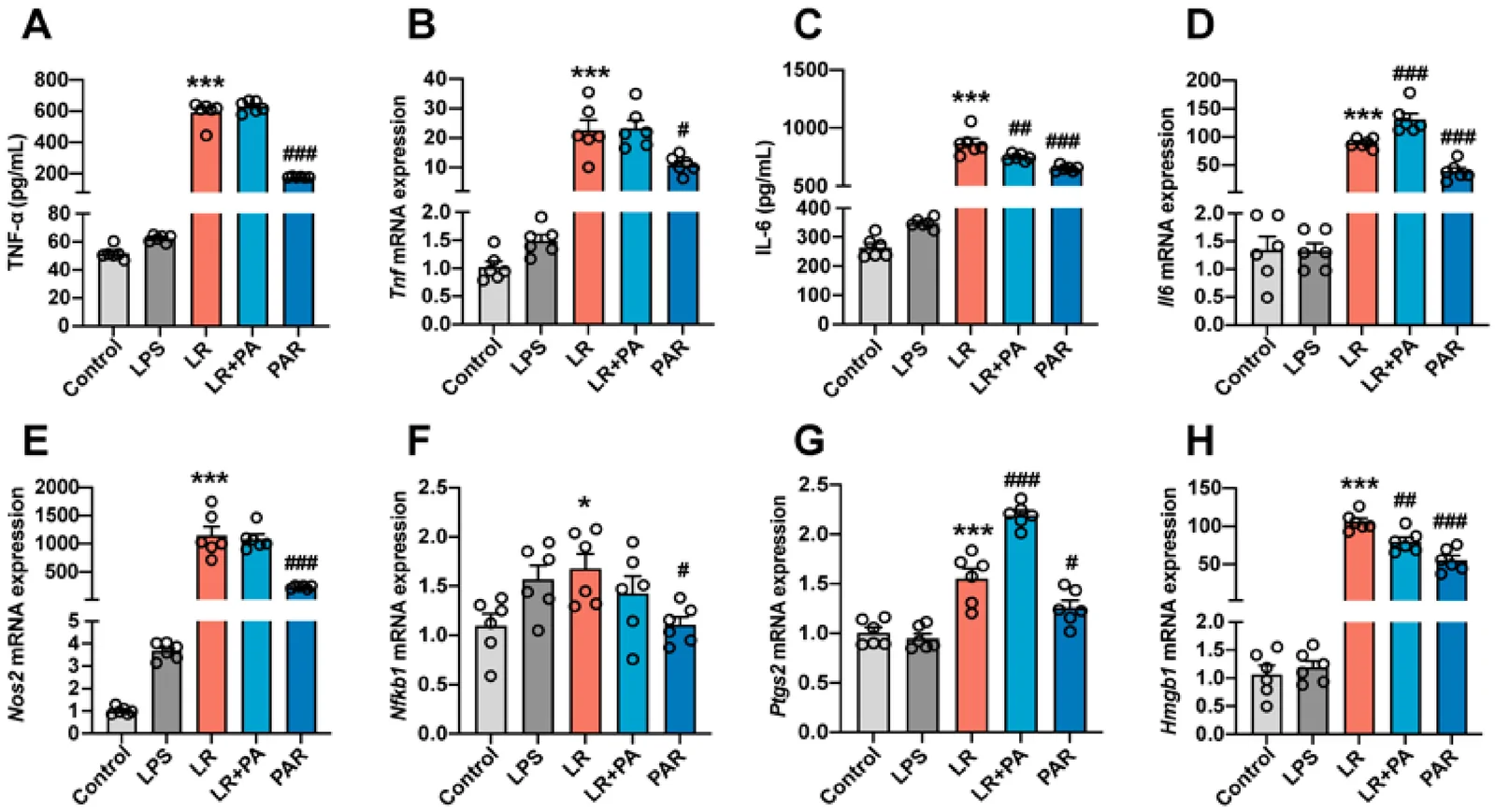
PA indirectly alleviated inflammatory responses in AML12 cells by regulating RAW264.7 cells. (**A**,**B**) TNF-α levels and *Tnf* mRNA expression in AML12 cells. (**C**,**D**) IL-6 levels and *Il6* mRNA expression in AML12 cells. (**E**–**H**) mRNA expression levels of *Nos2*, *Nfkb1*, *Ptgs2* and *Hmgb1* in AML12 cells. Circles represent individual data points. Data are presented as mean ± SEM, *n* = 6, * *p* < 0.05, *** *p* < 0.001 vs. control group; # *p* < 0.05, ## *p* < 0.01, ### *p* < 0.001 vs. LR group.

Compared with direct LPS stimulation, exposure to the supernatant from LPS-treated RAW264.7 cells resulted in higher TNF-α and IL-6 levels in AML12 cells (Figure 4A,C). In addition, the PAR group showed lower TNF-α and IL-6 levels than the LR + PA group. Similar changes were observed at the mRNA level. Compared with direct LPS stimulation, the supernatant from LPS-treated RAW264.7 cells increased the expression of *Tnf*, *Il6*, *Nos2*, *Nfkb1*, *Ptgs2* and *Hmgb1* in AML12 cells. The expression levels of these genes were generally lower in the PAR group than in the LR + PA group (Figure 4B,D–H).

### 3.5. PA Alleviated LPS-Induced Inflammation in RAW264.7 Cells

**Figure 5:**
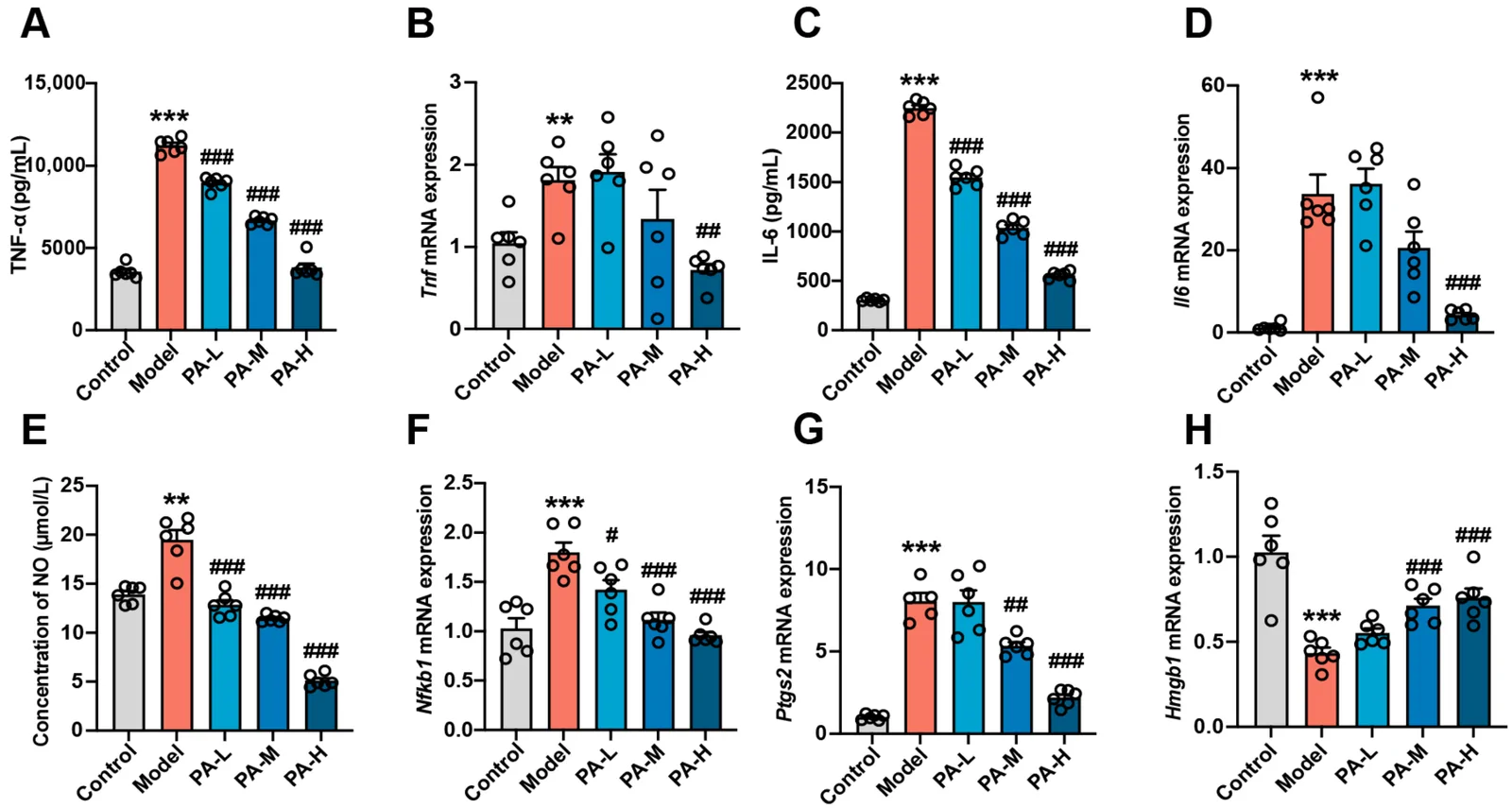
PA alleviated LPS-induced inflammatory responses in RAW264.7 cells. (**A**,**B**) TNF-α levels and *Tnf* mRNA expression. (**C**,**D**) IL-6 levels and *Il6* mRNA expression. (**E**) NO levels. (**F**–**H**) mRNA expression levels of *Nfkb1*, *Ptgs2* and *Hmgb1*. Circles represent individual data points. Data are presented as mean ± SEM, *n* = 6, ** *p* < 0.01, *** *p* < 0.001 vs. control group; # *p* < 0.05, ## *p* < 0.01, ### *p* < 0.001 vs. model group.

PA treatment at concentrations of 0.05–1 mmol/L for 24 h did not significantly affect RAW264.7 cell viability compared with the untreated control group (Figure S10A). Following LPS stimulation, TNF-α, IL-6 and NO levels in the cell supernatant were markedly increased in the model group and reduced following PA treatment (Figure 5A,C,E). Consistent with these changes, LPS markedly elevated the mRNA expression levels of *Tnf*, *Il6*, *Nfkb1* and *Ptgs2*, whereas PA attenuated these elevations. Additionally, *Hmgb1* expression was decreased in the model group and increased following PA treatment (Figure 5B,D,F–H).

### 3.6. PA Regulated Mitochondrial Function in RAW264.7 Cells

**Figure 6:**
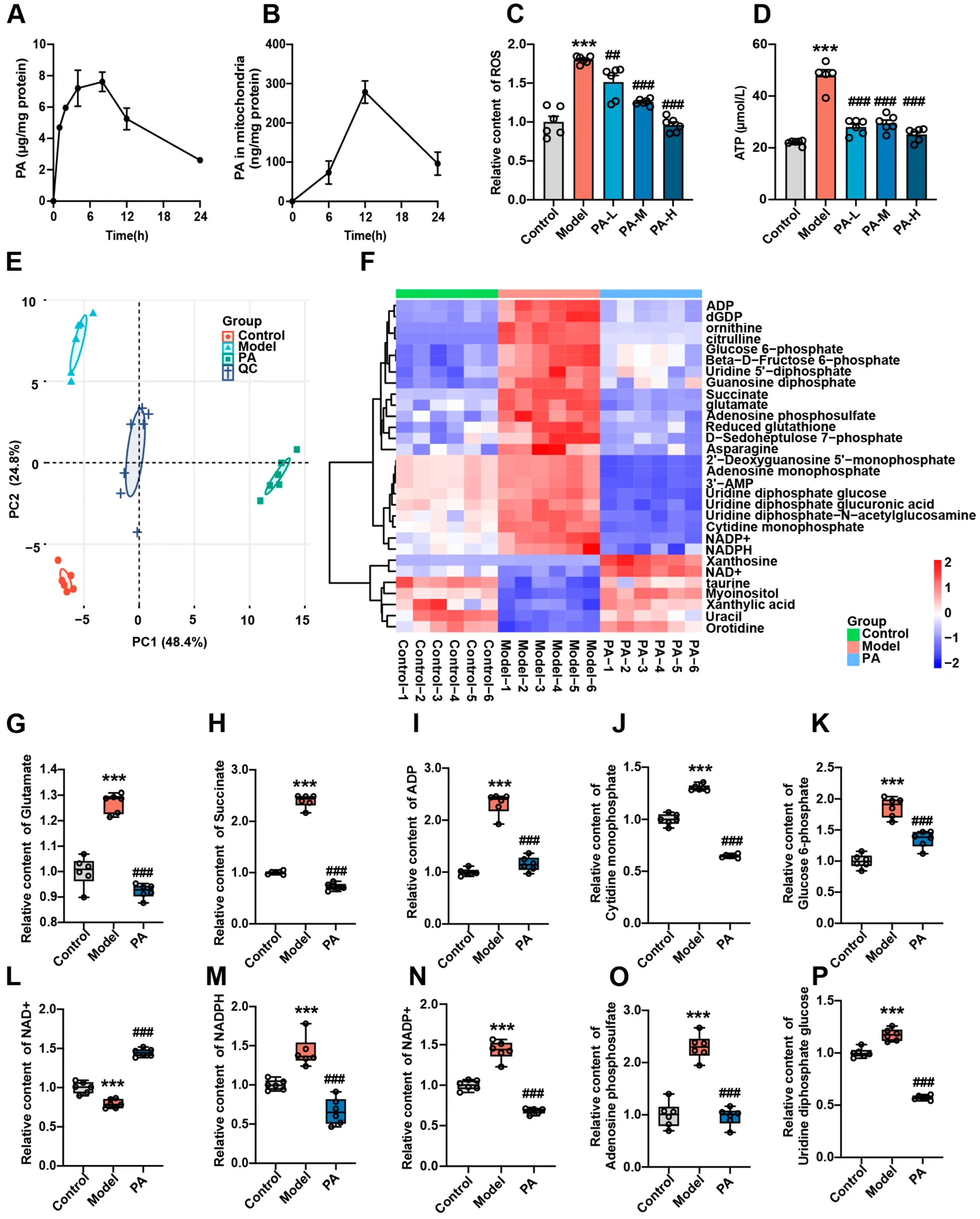
PA was distributed in the mitochondria of RAW264.7 cells and alleviated LPS-induced mitochondrial dysfunction. (**A**) Intracellular concentration-time profile of PA in RAW264.7 cells. (**B**) Mitochondrial distribution of PA in RAW264.7 cells. Data are presented as the mean ± SEM (*n* = 3). (**C**,**D**) ROS and ATP levels in RAW264.7 cells. (**E**) PCA score plot of cellular metabolomic profiles from the control, model and PA groups. (**F**) Heatmap of differential metabolites among the three groups. (**G**–**P**) Relative levels of representative differential metabolites. Circles represent individual data points. Data are presented as the mean ± SEM (*n* = 6). *** *p* < 0.001 vs. control group; ## *p* < 0.01, ### *p* < 0.001 vs. model group.

Following PA administration to RAW264.7 cells, the intracellular concentration-time profile of PA was determined (Figure 6A), with a C_max_ of 7.9 μg/mg and a T_max_ of 6.7 h (Table 2). PA was also detected in mitochondrial, nuclear and cytoplasmic fractions, with the highest levels observed at 12 h after administration (Figure 6B and Figure S3). In RAW264.7 cells, ROS levels were increased following LPS stimulation and reduced by PA treatment (Figure 6C). LPS stimulation also decreased mitochondrial membrane potential, as indicated by the reduced JC-1 red/green fluorescence ratio in mitochondria isolated from RAW264.7 cells, whereas PA treatment increased this ratio, with significant increases observed in the PA-M and PA-H groups (Figure S10B). Following LPS stimulation, ATP levels in the model group increased markedly, while PA attenuated this increase (Figure 6D). In addition, consistent with the PCR results in the livers of ALI mice, *Nfe2l2* expression was decreased in LPS-stimulated RAW264.7 cells and increased following PA treatment (Figure S4).

PCA of the cellular metabolomic data showed clear separation among the groups, with the PA group shifting toward the control group. The QC samples clustered closely in the center of the PCA plot, indicating good analytical stability and reliability (Figure 6E). Heatmap analysis showed distinct changes in differential metabolites among the control, model and PA groups (Figure 6F). Further analysis showed alterations in several metabolic pathways, including purine metabolism, glutathione metabolism, pyrimidine metabolism, and the TCA cycle (Figure S5). The levels of several differential endogenous metabolites, including glutamate, succinate, ADP, NADPH and NADP^+^, were notably increased following LPS stimulation, and these increases were attenuated by PA administration. NAD^+^ levels were decreased in LPS-stimulated cells and increased following PA administration (Figure 6G–P). Consistent with this finding, the NAD^+^/NADH ratio was significantly decreased in the model group compared with the control group, whereas PA-H treatment significantly restored the NAD^+^/NADH ratio (Figure S10C).

### 3.7. PA Metabolites Showed Potential Interactions with NDUFS7 of Mitochondrial Complex I

**Figure 7:**
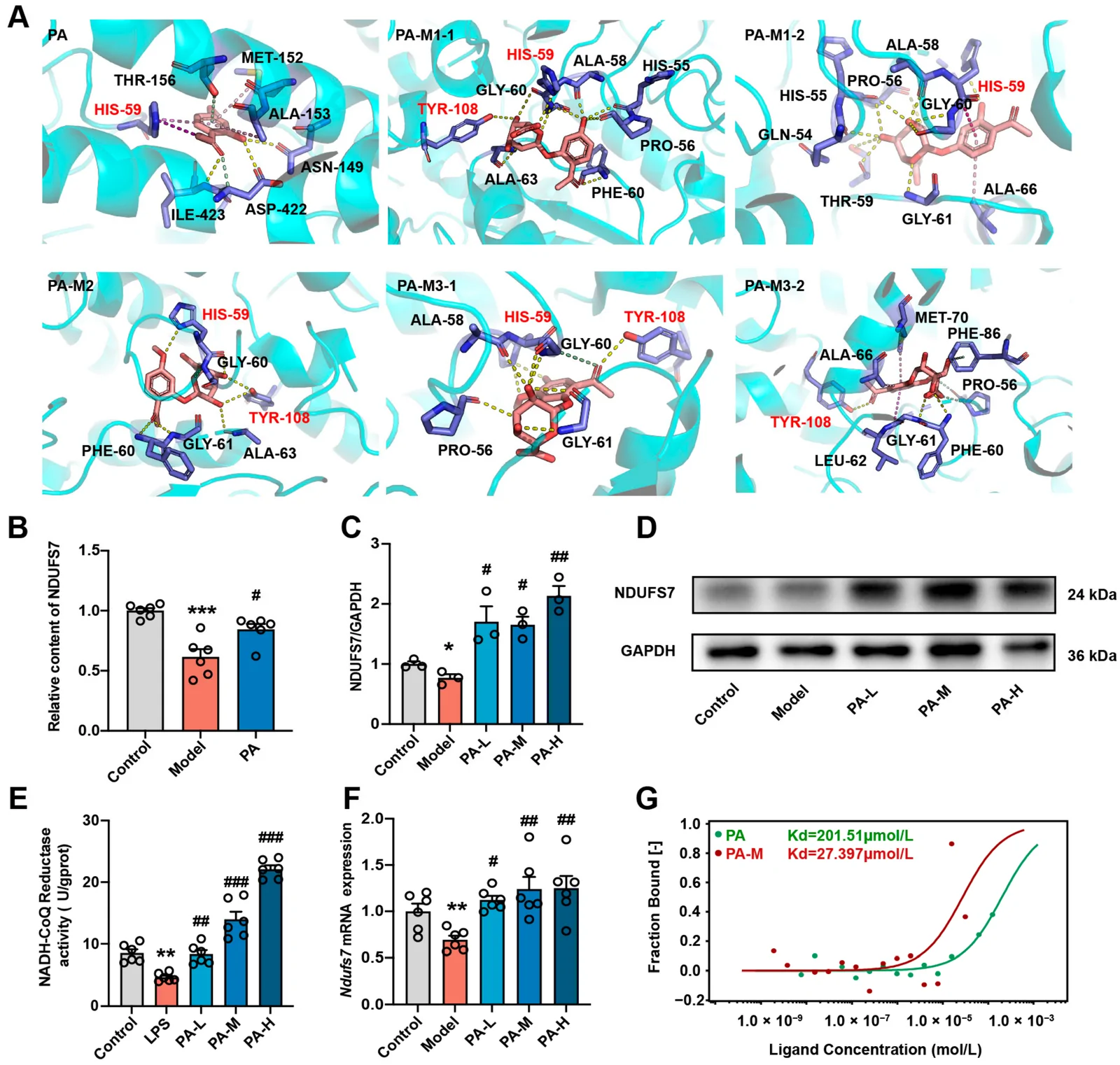
Effects of PA on NDUFS7 and mitochondrial complex I and their potential interactions with PA metabolites. (**A**) Molecular docking of PA and its metabolites with mitochondrial complex I. (**B**) Proteomic analysis of NDUFS7 expression in the livers of ALI mice (*n* = 6). (**C**,**D**) Representative Western blot and quantitative analysis of NDUFS7 expression (*n* = 3). (**E**,**F**) Mitochondrial complex I activity and *Ndufs7* mRNA expression in RAW264.7 cells (*n* = 6). (**G**) Interactions of PA and its metabolites with NDUFS7 determined by MST analysis. Circles in (**B**,**C**,**E**,**F**) represent individual data points, while the colored circles in (**G**) represent the experimental MST data points. Data in (**B**–**F**) are presented as mean ± SEM. * *p* < 0.05, ** *p* < 0.01, *** *p* < 0.001 vs. control group; # *p* < 0.05, ## *p* < 0.01, ### *p* < 0.001 vs. model group. Different colored dashed lines in (**A**) indicate different molecular interactions: yellow, conventional hydrogen bonds; green, carbon-hydrogen bonds and Pi-donor hydrogen bonds; light pink, Pi-alkyl interactions; hot pink, amide-Pi stacking; violet, Pi-Pi T-shaped interactions; and purple, Pi-sigma interactions.

PA was metabolized into several metabolites in human liver microsomes (HLM) and primary human hepatocytes (PHH) (Figure S6). Among them, the glucuronide conjugates PA-M1, PA-M2 and PA-M3 were major metabolites in PHH, with five possible structures considered for subsequent analysis (Table S1). Molecular docking analysis showed that the five possible metabolite structures had lower predicted binding energies with mitochondrial complex I than PA, with potential interactions involving HIS59 and TYR108 (Figure 7A and Figure S7, Table 3).

Molecular docking further indicated potential interactions of PA metabolites with the NDUFS7 and NDUFS2 subunits of mitochondrial complex I. The expression levels of NDUFS7 and NDUFS2 were markedly decreased in the livers of ALI mice. PA treatment increased NDUFS7 expression, while no obvious change was observed in NDUFS2 expression (Figure 7B–D and Figure S8). In RAW264.7 cells, mitochondrial complex I activity was increased following PA treatment, accompanied by an increase in *Ndufs7* mRNA expression (Figure 7E,F). Based on these observations, NDUFS7 was selected for further MST analysis. MST analysis showed that PA metabolites had lower Kd values for NDUFS7 than PA (Figure 7G).

## 4. Discussion

ALI is characterized by acute impairment of liver function and is associated with poor prognosis and high mortality. Common causes include viral hepatitis, bacterial infections, and certain drugs. Previous studies have suggested that inflammatory responses and oxidative stress play important roles in ALI [23], and these two processes are closely interconnected. Mitochondria-derived ROS can activate inflammatory signaling in hepatocytes and macrophages, while inflammatory mediators may in turn impair mitochondrial antioxidant capacity, potentially contributing to a self-amplifying cycle of liver damage. The LPS/D-GalN-induced liver injury model has been widely used to investigate the pathogenesis of ALI and potential therapeutic interventions [24]. As a typical endotoxin, LPS can stimulate macrophages to release inflammatory mediators and activate multiple inflammatory signaling pathways. D-GalN is a hepatotoxic agent that disrupts hepatic glucose and phospholipid metabolism, thereby increasing susceptibility to LPS-induced liver injury [25].

PA is a phenolic compound found in the root bark of *Paeonia suffruticosa* Andr. and has been reported to possess various biological activities. PA has been investigated in osteoarthritis, skin inflammation, diabetic encephalopathy, cerebral ischemic injury, and myocardial ischemia/reperfusion-associated injuries. In addition, previous studies have shown that PA can mitigate injuries in kidney, lung and liver [15]. However, evidence regarding the effects of PA on LPS/D-GalN-induced ALI remains limited.

In this study, PA reduced serum ALT and AST levels and alleviated inflammatory infiltration and hepatic hemorrhage. PA also reduced the secretion and expression of pro-inflammatory cytokines in liver tissue. In addition, *Nfkb1* expression was increased following LPS/D-GalN challenge and decreased following PA treatment. Previous studies have shown that NF-κB can regulate *Ptgs2* expression and affect downstream inflammatory pathways [26]. As a nuclear transcription factor, NF-κB participates in various biological processes, including inflammation and apoptosis. Activation of the NF-κB signaling pathway can induce the expression of multiple inflammatory cytokines. These findings suggest that the protective effects of PA against ALI may be associated with the attenuation of inflammatory responses (Figure 1).

Proteomic analysis was further used to explore the potential processes associated with the effects of PA in ALI. Differential protein analysis showed that PA mainly affected oxidative phosphorylation, cellular respiration as well as the generation of precursor metabolites and energy in ALI mice. The differential proteins were mainly enriched in mitochondrial components and were associated with transporter activity. PPI analysis also highlighted changes in proteins related to mitochondrial and ribosomal functions (Figure 2). Similarly, Metascape analysis showed marked changes in metabolic processes, with enrichment mainly associated with the liver and bone marrow (Figure S1). These findings highlighted the potential involvement of mitochondria in ALI and the effects of PA, consistent with previous studies [27]. In addition, previous studies have highlighted the involvement of bone marrow-derived macrophages in hepatic inflammation during ALI [28]. Taken together, these findings prompted us to further investigate the potential involvement of mitochondrial function and macrophages in the effects of PA on ALI.

Mitochondria are essential for maintaining intracellular homeostasis and are closely involved in various pathological processes. The mitochondrial electron transport chain, which consists of complexes I-V in the inner mitochondrial membrane, links nutrient oxidation to ATP production through oxidative phosphorylation. Mitochondrial complex I catalyzes the oxidation of NADH and transfers electrons to ubiquinone, while coupling electron transfer to proton translocation for ATP production [29]. Mitochondrial complex I has also been implicated in innate immunity and bone homeostasis. Loss of the NDUFS4 subunit can trigger systemic inflammation as well as bone sclerosis, and shift hepatic metabolism toward glycolysis. In addition, mitochondrial dysfunction can lead to excessive ROS production and macrophage activation, further aggravating the inflammatory response [30].

In our research, molecular docking analysis suggested more favorable predicted interactions of several PA metabolites with mitochondrial complex I compared with the parent compound. Combined with the quantitative proteomics results, NDUFS7 emerged as a potential candidate subunit associated with the effects of PA. The MST assay further showed lower Kd values for PA metabolites with recombinant NDUFS7 than for PA (Figure 7). Taken together, these findings support the potential involvement of NDUFS7 in the effects of PA and its metabolites on mitochondrial function, rather than establishing NDUFS7 as a causal mediator. It should also be noted that the MST assay was performed using isolated recombinant NDUFS7 rather than NDUFS7 assembled within native mitochondrial complex I. Therefore, although the MST results provide preliminary biophysical evidence supporting interactions of PA and its metabolites with recombinant NDUFS7 under cell-free assay conditions, they do not fully recapitulate the native structural environment of NDUFS7 within intact mitochondrial complex I. Further studies in the context of intact mitochondrial complex I are required to establish the physiological relevance of these interactions.

Metabolic alterations are common features of liver disease, and cellular metabolism plays an important role in maintaining liver homeostasis and regeneration. Metabolomic analysis further showed that PA mainly affected alanine, aspartate and glutamate metabolism, pyrimidine metabolism, the TCA cycle and riboflavin metabolism (Figure 3). Amino acid metabolism is essential for maintaining normal cellular function. Glutamate links carbohydrate and amino acid metabolism through the TCA cycle [31]. Studies have shown that mitochondrial dysfunction is accompanied by alterations in pyruvate and alanine metabolism [32]. Mitochondrial aspartate synthesis is also important for cell proliferation and may therefore be relevant to liver repair and regeneration during ALI [33]. In addition, inhibition of cytosolic pyrimidine metabolism has been reported to trigger mitochondrial DNA release into the cytoplasm and activate innate immunity [34].

TCA cycle metabolites can also influence cellular and immune responses. Metabolites such as acetyl-CoA, itaconate, succinate, and fumarate have been implicated in the regulation of immune responses, while succinate can also function as a systemic metabolic signal [35]. Although the specific roles of these metabolites in ALI remain unclear, the observed metabolic alterations suggest a potential association between the effects of PA and metabolic regulation during ALI. Riboflavin, a B vitamin required for respiratory-chain flavoenzymes [36], has also been reported to support electron transport chain function under conditions of mitochondrial oxidative phosphorylation dysfunction [37]. Together with our proteomic findings, these metabolic changes suggest that the effects of PA may be associated with mitochondrial energy metabolism and related metabolic processes.

Mitochondrial complex II oxidizes succinate to fumarate and transfers electrons to ubiquinone [38]. In our study, fumarate and FAD levels were increased in ALI mice and decreased following PA administration (Figure S9). Complexes III and IV are involved in mitochondrial ROS production and respiratory regulation during inflammation, while complex V drives ATP synthesis from ADP using an electrochemical gradient [39,40]. Our results showed alterations in ATP and ADP levels in LPS-stimulated RAW264.7 cells following PA treatment (Figure 6D,I). Together, these findings suggest broader changes in mitochondrial energy metabolism following PA treatment. However, the direct effects of PA and its metabolites on other mitochondrial respiratory-chain complexes remain unclear and require further investigation.

Acute liver failure is characterized by substantial loss of hepatic parenchymal cells followed by regenerative responses [41]. In subsequent experiments, AML12 cells were used to further investigate the effects of PA on hepatocyte inflammatory responses. Previous studies have suggested that hepatocytes exhibit pronounced inflammatory responses following exposure to inflammatory mediators [42]. In our study, supernatants from LPS-stimulated RAW264.7 cells induced greater inflammatory responses in AML12 cells than direct LPS stimulation, while these responses were attenuated when PA was administered to RAW264.7 cells (Figure 4). These findings suggest that macrophages may contribute to the effects of PA on inflammatory responses in hepatocytes.

In RAW264.7 cells, PA reduced inflammatory responses and cellular ROS levels, with an improvement in mitochondrial membrane potential. Mitochondria are closely involved in cellular redox homeostasis, and mitochondrial dysfunction can contribute to ROS accumulation and inflammatory responses in macrophages [43]. Nrf2 is an important regulator of cellular redox homeostasis [44]. The changes in *Nfe2l2* expression observed in our study (Figure S4) were consistent with the changes in oxidative stress following PA treatment [45,46]. In addition, PA was detected in the mitochondrial fraction, accompanied by changes in *Ndufs7* expression, mitochondrial complex I activity, and energy-related metabolic pathways. Together with the docking and MST results, these findings suggest a potential association among PA and its metabolites, NDUFS7, and mitochondrial function in inflammatory macrophages. However, further functional studies targeting NDUFS7 are required to determine whether NDUFS7 directly contributes to these effects.

In summary, our findings suggest that PA alleviates LPS/D-GalN-induced ALI, with its effects associated with the regulation of inflammatory responses and mitochondrial function. Multi-omics and cellular analyses further highlighted macrophages and mitochondrial energy metabolism as potentially relevant to the effects of PA. NDUFS7 emerged as a potential candidate associated with PA and its metabolites, although its causal role remains to be established. These findings provide a basis for further investigation of the protective mechanisms of PA in ALI.

## 5. Conclusions

PA alleviated LPS/D-GalN-induced acute liver injury in addition to reducing inflammatory responses and improving mitochondrial function. Multi-omics and cellular analyses further suggested the potential involvement of macrophage mitochondrial function, with NDUFS7 emerging as a candidate associated with PA and its metabolites. These findings provide new insights into the protective effects of PA against acute liver injury, although the causal role of NDUFS7 requires further investigation.

## Figures and Tables

**Table 1:** Nucleotide sequences of PCR primers used in this study.

Genes	5′-3′ Primer Sequences		Accession Number
*Il6*	Forward	TAGTCCTTCCTACCCCAATTTCC	NM_031168
	Reverse	TTGGTCCTTAGCCACTCCTTC	
*Il1b*	Forward	GAAATGCCACCTTTTGACAGTG	NM_008361
	Reverse	TGGATGCTCTCATCAGGACAG	
*Nfkb1*	Forward	ATGGCAGACGATGATCCCTAC	NM_008689
	Reverse	TGTTGACAGTGGTATTTCTGGTG	
*Ptgs2*	Forward	TTCAACACACTCTATCACTGGC	NM_011198
	Reverse	AGAAGCGTTTGCGGTACTCAT	
*Nfe2l2*	Forward	TCTTGGAGTAAGTCGAGAAGTGT	NM_010902
	Reverse	GTTGAAACTGAGCGAAAAAGGC	
*Hmgb1*	Forward	GCTGACAAGGCTCGTTATGAA	NM_010439
	Reverse	CCTTTGATTTTGGGGCGGTA	
*Tnf*	Forward	CTGAACTTCGGGGTGATCGG	NM_013693
	Reverse	GGCTTGTCACTCGAATTTTGAGA	
*Nos2*	Forward	GGAGTGACGGCAAACATGACT	NM_001313922
	Reverse	TCGATGCACAACTGGGTGAAC	
*Ndufs7*	Forward	ACCACTACTCCTACTCGGTTG	NM_029272
	Reverse	GTTCACGCTTGATCTTCCGTT	
*actb*	Forward	ACGGCCAGGTCATCACTATTG	NM_007393
	Reverse	AGGGGCCGGACTCATCGTA	

**Table 2:** Pharmacokinetic parameters of PA in RAW264.7 cells.

Parameters	C_max_ (μg/mg)	T_max_ (h)
$\bar{x}$	7.9	6.7
s	1.5	2.3

**Table 3:** Binding energies of PA and its metabolites with mitochondrial complex I.

PA and Metabolites	Binding Energies
PA	−75.00
PA-M1-1	−128.19
PA-M1-2	−109.22
PA-M2	−118.18
PA-M3-1	−121.82
PA-M3-2	−106.87

## Data Availability

The data supporting the findings of this study are available within the article and its Supplementary Materials. Additional data are available from the corresponding author upon reasonable request.

## Supplementary Materials

The following supporting information can be downloaded at: https://www.mdpi.com/article/10.3390/antiox15091079/s1, Figure S1: Proteomic analysis based on the Metascape database. (A) PA notably affected the generation of precursor metabolites and energy-related processes in the livers of ALI mice. (B) Differentially expressed proteins were mainly enriched in metabolic processes. (C) Tissue enrichment analysis of differential proteins was mainly associated with the liver and bone marrow; Figure S2: Effects of PA on mitochondria-related proteins in the livers of ALI mice. Relative levels of selected mitochondria-related proteins identified by proteomic analysis. Data are presented as mean ± SEM (n = 6). * *p* < 0.05, ** *p* < 0.01, *** *p* < 0.001 vs. control group; # *p* < 0.05, ## *p* < 0.01, ### *p* < 0.001 vs. model group; Figure S3: Distribution of PA in the nucleus and cytoplasm of RAW264.7 cells. (A) Distribution of PA in the nuclear fraction of RAW264.7 cells within 12 h after treatment. (B) Distribution of PA in the cytoplasmic fraction of RAW264.7 cells within 12 h after treatment. Data are presented as mean ± SEM (n = 3); Figure S4: Effects of PA on Nfe2l2 mRNA expression. (A, B) Relative mRNA expression of Nfe2l2 in LPS-stimulated RAW264.7 cells and the livers of ALI mice following PA treatment. Data are presented as mean ± SEM (n = 6). * *p* < 0.05, ** *p* < 0.01, *** *p* < 0.001 vs. control group; # *p* < 0.05, ## *p* < 0.01, ### *p* <0.001 vs. model group; Figure S5: Effects of PA on metabolic pathways in LPS-stimulated RAW264.7 cells. (A) Differential metabolic pathways identified by pathway analysis. (B) Differential metabolic pathways identified by enrichment analysis; Figure S6: Putative metabolic pathways of PA in human liver microsomes and primary human hepatocytes; Figure S7: Predicted interactions of PA and its proposed metabolite structures with mitochondrial complex I. (A) Predicted interaction of PA with HIS59. (B–F) Predicted interactions of PA-M1-1, PA-M1-2, PA-M2, PA-M3-1, and PA-M3-2 with residues in the defined binding site of mitochondrial complex I; Figure S8: Effects of PA on NDUFS2 expression in the livers of ALI mice. PA treatment did not significantly reverse the decrease in NDUFS2 expression in ALI mice. Data are presented as mean ± SEM (n = 6). * *p* < 0.05, ** *p* < 0.01, *** *p* < 0.001 vs. control group; # *p* < 0.05, ## *p* < 0.01, ### *p* < 0.001 vs. model group; Figure S9: Effects of PA on fumarate and FAD levels in ALI mice. (A, B) Relative levels of fumarate and FAD following PA treatment. Data are presented as mean ± SEM (n = 6). * *p* < 0.05, ** *p* < 0.01, *** *p* < 0.001 vs. control group; # *p* < 0.05, ## *p* < 0.01, ### *p* < 0.001 vs. model group; Figure S10: Effects of PA on RAW264.7 cell viability and mitochondrial function, with assessment of molecular docking. (A) Effects of PA on RAW264.7 cell viability. (B) Effects of PA on mitochondrial membrane potential in LPS-stimulated RAW264.7 cells, as determined by the JC-1 red/green fluorescence ratio. (C) Effects of PA on the NAD^+^/NADH ratio in LPS-stimulated RAW264.7 cells. (D) Comparison of the predicted binding pose of re-docked HQH with native HQH in the original 6ZTQ structure. Data in (A–C) are presented as mean ± SEM (n = 6). ** *p* < 0.01, *** *p* < 0.001 vs. control group; ### *p* < 0.001 vs. model group; Table S1: UPLC/Q-TOF MS information of PA metabolites in primary human hepatocytes and human liver microsomes; Supplementary Methods: Liquid chromatography and mass spectrometry conditions for proteomic and metabolomic analyses.

## Abbreviations

The following abbreviations are used in this manuscript:

ALI	Acute liver injury
ALT	Alanine aminotransferase
AST	Aspartate aminotransferase
ATP	Adenosine triphosphate
COX-2	Cyclooxygenase-2
D-GalN	D-galactosamine
FAD	Flavin adenine dinucleotide
GO	Gene Ontology
HLM	Human liver microsomes
IL-1β	Interleukin-1 beta
IL-6	Interleukin-6
iNOS	Inducible nitric oxide synthase
KEGG	Kyoto Encyclopedia of Genes and Genomes
LPS	Lipopolysaccharide
MDA	Malondialdehyde
MST	Microscale thermophoresis
NDUFS7	NADH dehydrogenase [ubiquinone] iron-sulfur protein 7
NO	Nitric oxide
OPLS-DA	Orthogonal partial least-squares discriminant analysis
PA	Paeonol
PCA	Principal component analysis
PHH	Primary human hepatocytes
PLS-DA	Partial least-squares discriminant analysis
PPI	Protein–protein interaction
QC	Quality control
ROS	Reactive oxygen species
TCA cycle	Tricarboxylic acid cycle
TNF-α	Tumor necrosis factor-alpha
VIP	Variable importance in projection
