# Supplementary material for: Paeonol and Its Metabolites Alleviate LPS/D-GalN-Induced Acute Liver Injury in Mice: Potential Involvement of NDUFS7 and Macrophage Mitochondrial Function
Source: Antioxidants (Basel). 2026 Aug 28;15(9):1079. doi: 10.3390/antiox15091079 (PMC13603529; doi:10.3390/antiox15091079)
Supplement: Supplementary file 1 [file antioxidants-15-01079-s001.zip › antioxidants-4506781-supplementary.pdf]

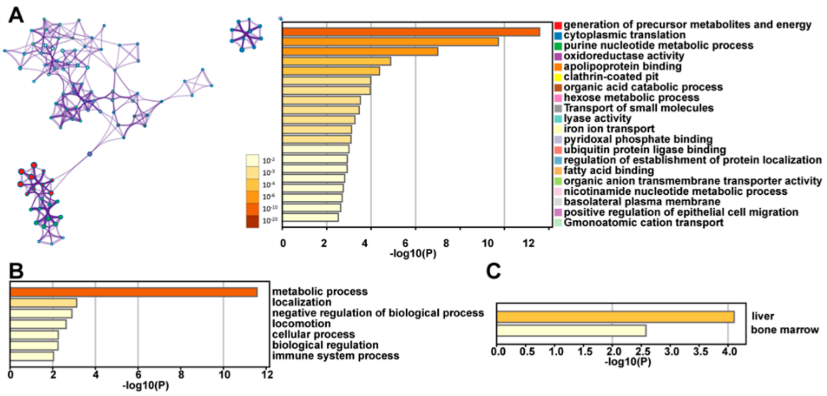

Figure S1. Proteomic analysis based on the Metascape database. (A) PA notably affected the generation of precursor metabolites and energy-related processes in the livers of ALI mice. (B) Differentially expressed proteins were mainly enriched in metabolic processes. (C) Tissue enrichment analysis of differential proteins was mainly associated with the liver and bone marrow.

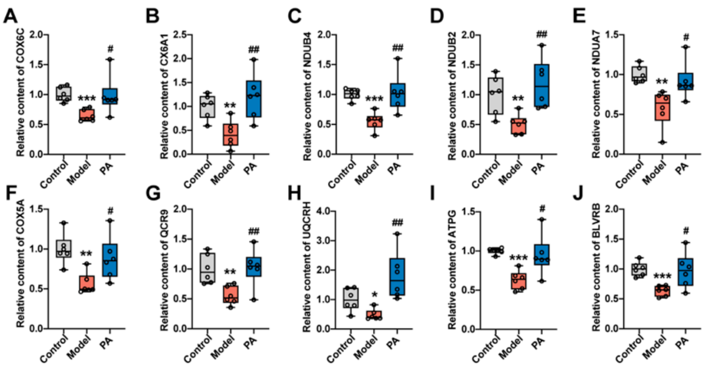

Figure S2. Effects of PA on mitochondria-related proteins in the livers of ALI mice. Relative levels of selected mitochondria-related proteins identified by proteomic analysis. Data are presented as mean ± SEM (n = 6). *P<0.05, **P<0.01, ***P<0.001 vs. control group; #P<0.05, ##P<0.01, ###P<0.001 vs. model group.

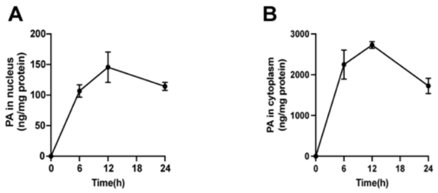

Figure S3. Distribution of PA in the nucleus and cytoplasm of RAW264.7 cells. (A) Distribution of PA in the nuclear fraction of RAW264.7 cells within 12 h after treatment. (B) Distribution of PA in the cytoplasmic fraction of RAW264.7 cells within 12 h after treatment. Data are presented as mean ± SEM (n = 3).

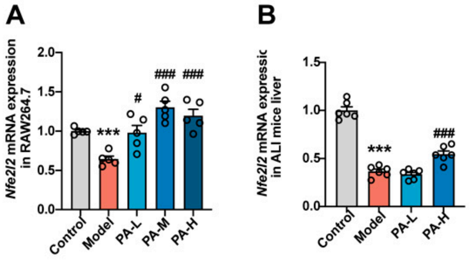

Figure S4. Effects of PA on *Nfe2l2* mRNA expression. (A, B) Relative mRNA expression of *Nfe2l2* in LPS-stimulated RAW264.7 cells and the livers of ALI mice following PA treatment. Data are presented as mean ± SEM (n = 6). *P<0.05, **P<0.01, ***P<0.001 vs. control group; #P<0.05, ##P<0.01, ###P<0.001 vs. model group.

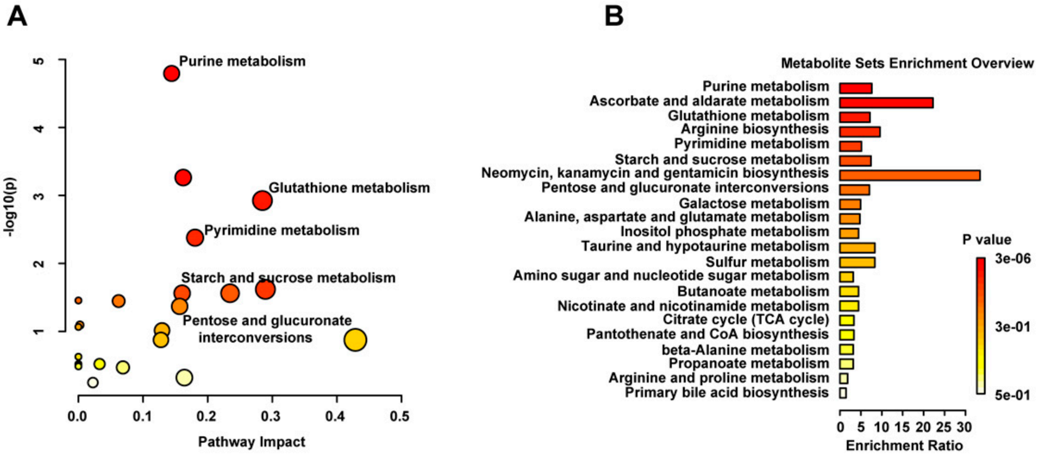

Figure S5. Effects of PA on metabolic pathways in LPS-stimulated RAW264.7 cells. (A) Differential metabolic pathways identified by pathway analysis. (B) Differential metabolic pathways identified by enrichment analysis.

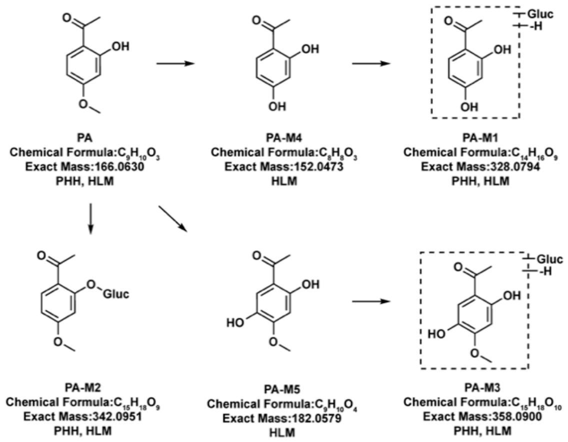

Figure S6. Putative metabolic pathways of PA in human liver microsomes and primary human hepatocytes.

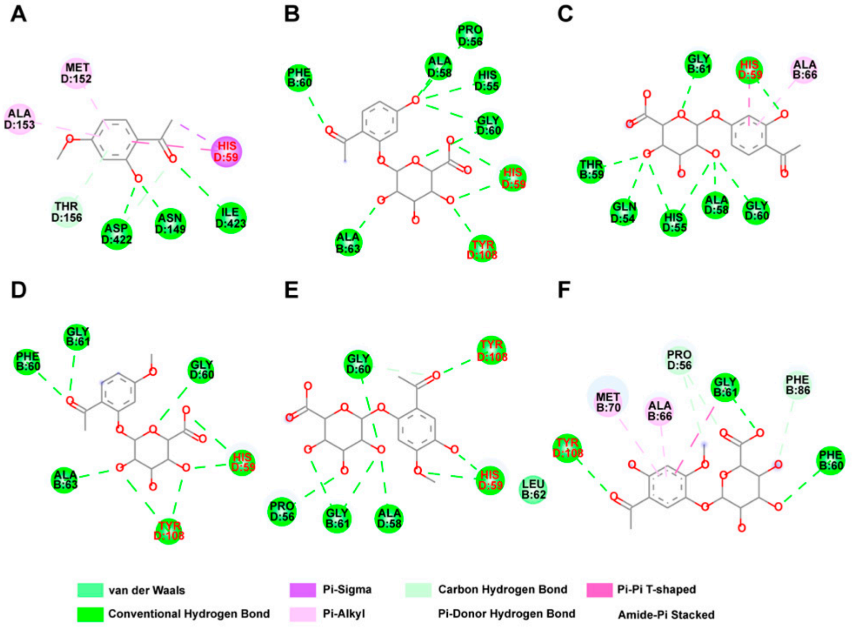

Figure S7. Predicted interactions of PA and its proposed metabolite structures with mitochondrial complex I. (A) Predicted interaction of PA with HIS59. (B–F) Predicted interactions of PA-M1-1, PA-M1-2, PA-M2, PA-M3-1, and PA-M3-2 with residues in the defined binding site of mitochondrial complex I.

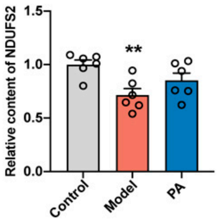

Figure S8. Effects of PA on NDUFS2 expression in the livers of ALI mice. PA treatment did not significantly reverse the decrease in NDUFS2 expression in ALI mice. Data are presented as mean ± SEM (n = 6). *P<0.05, **P<0.01, ***P<0.001 vs. control group; #P<0.05, ##P<0.01, ###P<0.001 vs. model group.

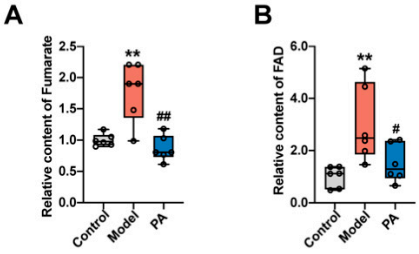

Figure S9. Effects of PA on fumarate and FAD levels in ALI mice. (A, B) Relative levels of fumarate and FAD following PA treatment. Data are presented as mean ± SEM (n = 6). *P<0.05, **P<0.01, ***P<0.001 vs. control group; #P<0.05, ##P<0.01, ###P<0.001 vs. model group.

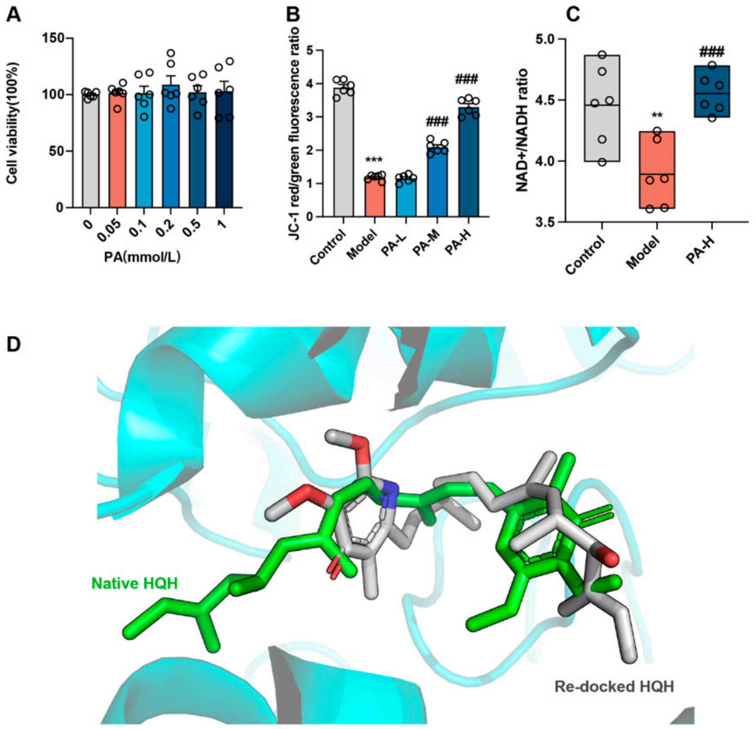

Figure S10. Effects of PA on RAW264.7 cell viability and mitochondrial function, with assessment of molecular docking. (A) Effects of PA on RAW264.7 cell viability. (B) Effects of PA on mitochondrial membrane potential in LPS-stimulated RAW264.7 cells, as determined by the JC-1 red/green fluorescence ratio. (C) Effects of PA on the NAD^+/NADH ratio in LPS-stimulated RAW264.7 cells. (D) Comparison of the predicted binding pose of re-docked HQH with native HQH in the original 6ZTQ structure. Data in (A–C) are presented as mean ± SEM (n = 6). $^{**}P < 0.01$, $^{***}P < 0.001$ vs. control group; ###$P < 0.001$ vs. model group.

Table S1. UPLC/Q-TOF MS information of PA metabolites in primary human hepatocytes and human liver microsomes.

Parents & Metabolites	tR/min	Molecular	Ion mode	Observed m/z	Calculated m/z	Error	Main fragments m/z	Metabolite Description	LC-MS/MS Peak Area		
									PHH	Supernatant	HLM
PA	13.83	C9H10O3	[M-H]-	165.0544	165.0552	4.8	150.0322;135.0093;122.0362;91.0189	Prototype drug	3.0E+3	8.6E+3	1.6E+4
PA-M1	12.51	C14H16O9	[M-H]-	327.0697	327.0716	5.8	327.0697;151.0401;135.0097	Demethylation + Glucuronic acid binding	9.4E+3	2.2E+4	6.7E+5
PA-M2	12.73	C15H18O9	[M-H]-	341.0884	341.0873	3.2	341.0884;165.0560;150.0319;135.0091;122.0373	Glucuronic acid binding	2.7E+4	9.2E+3	1.5E+6
PA-M3	12.73	C15H18O10	[M-H]-	357.0821	357.0822	0.28	181.0508	Single oxidation + Glucuronic acid binding	4.0E+3	2.0E+4	2.9E+5
PA-M4	12.46	C8H8O3	[M-H]-	151.0399	151.0395	2.6	151.0399;135.0081;109.0293;91.0180	Demethylation	ND	ND	1.4E+4
PA-M5	15.35	C9H10O4	[M-H]-	181.0505	181.0501	2.2	166.0262;122.0379	Single oxidation	ND	ND	1.8E+3

Supplementary Methods: Liquid chromatography and mass spectrometry conditions for proteomic and metabolomic analyses

1. Liquid Chromatography Conditions of Proteomics

Trap column: Eksigent ChromXP (C18, 75 μm × 2 cm, 3 μm, 120 Å); analytical column: Eksigent ChromXP (C18, 300 μm × 15 cm, 3 μm, 120 Å); column temperature: 40 °C; G1 aqueous phase (A): 0.1% formic acid-water; G1 organic phase (B): 0.1% formic acid-acetonitrile; G2 aqueous phase (A): 10% acetonitrile-water; G2 organic phase (B): 10% water-acetonitrile; flow rate: 5 μL/min; analysis time: 90 min; gradient elution: 0-0.1 min (5 % to 8 % B), 0.1-78 min (8 % to 35 % B), 78-80 min (35 % to 80 % B), 80-84 min (80 % B), 84-85 min (80 % to 5 % B), 85-90 min (5 % B).

2. Mass Spectrometry Conditions of Proteomics

SCIEX M5 MicroLC microfluidic liquid system combined with AB SCIEX TripleTOF 5600; Detection mode: positive ion mode; ISV: 5500 V, GS1: 12 psi, GS2: 20 psi, CUR: 30 psi, DP: 80 V, TEM: 330 ℃, accumulation time: 0.25 s, production scan: IDA: 40, m/z: 100-1500 Da, accumulation time: 0.05 s, Rolling CE: enabled, collision energy spread: 5, ion release delay: 68, ion release width: 26.

3. Liquid Chromatography Conditions of Metabolomics

Chromatographic column: Waters XBridge Amide 3.5 μm, 4.6 × 100 mm Column; column temperature: 40 °C; aqueous phase (A): 5 mM ammonium acetate in ultrapure water containing 5 % acetonitrile, adjusted to pH 9.0 with ammonia (Nanjing Reagent, China); organic phase (B): acetonitrile; flow rate: 0.4 mL/min; analysis time: 26.0 min; gradient elution: 0-3.0 min (85% B), 3.0-6.0 min (85% to 30% B), 6.0-15.0 min (30% to 2% B), 15.0-18.0 min (2% B), 18.0-19.0 min (2% to 85% B), 19.0-26.0 min (85% B).

4. Mass Spectrometry Conditions of Metabolomics

The instrument used was AB SCIEX TripleTOF 5600. Electrospray ionization (ESI) was used for MS detection in negative ion mode with the following parameters: TOF MS scan, m/z: 50-1000 Da; product ion scan, m/z: 50-900 Da; Gas 1, 50 psi; Gas 2, 30 psi; CUR, 30 psi; TEM, 500; ISVF, −4500 V; DP, −100 V; CE, −10 V; CE in production scan (IDA), -35 V.
